# External fixation-assisted reduction for the treatment of neglected hip dislocations with limb length discrepancy: a retrospective study of 13 cases

**DOI:** 10.1186/s12891-019-3015-0

**Published:** 2019-12-26

**Authors:** Pengyu Li, Fulin Tao, Wenhao Song, Jinlei Dong, Daodi Qiu, Dongsheng Zhou

**Affiliations:** Department of Orthopedic Surgery, Shandong Provincial Hospital Affiliated to Shandong University, 324 Jingwu Road, Jinan, Shandong China

**Keywords:** Neglected hip dislocation, Limb length discrepancy, External fixation

## Abstract

**Background:**

The purpose of this study was to evaluate a new method for treating neglected hip dislocation with limb length discrepancy by using external fixation-assisted pre-reduction.

**Methods:**

Thirteen patients admitted between January 2010 to February 2018 with a mean duration from injury to surgery of 5.0 ± 2.1 months and an average preoperative leg-length discrepancy of 7.7 ± 2.3 cm were enrolled in this study. The dislocation and associated acetabular fracture type, clinical outcomes and residual limb length discrepancy were evaluated.

**Results:**

All patients had posterior dislocations, and nine patients presented with acetabular fractures and were followed-up for at least 12 months. The average traction duration of external fixators was 28.8 ± 8.0 days and all patients received second-stage open reduction and internal fixation. Six patients showed residual limb length discrepancy within 2 cm. Patients showed significant improvement in hip function and pain relief. Complications including avascular femoral head necrosis and osteoarthritis occurred in three patients.

**Conclusion:**

Effective correction of limb length discrepancy and improved function were observed in patients with neglected hip dislocations and limb equality using traction by external fixation combined with second-stage open reduction. Further follow-up is required to determine long-term outcomes.

## Background

Traumatic hip dislocation is usually caused by high-energy trauma and accounts for 5% of all traumatic joint dislocations [1]. Most dislocations are caused by motor vehicle accidents and associated with unfastened safety belts [2]. Early recognition and a prompt reduction is of great importance [3, 4] because delayed diagnosis may lead to long-term complications including avascular necrosis of the femoral head (AVN) and osteoarthritis [5]. Neglected hip dislocations are rare in adults, especially in high-income countries [6]. However, in developing countries, patients may not seek medical attention or visit the hospital many days after trauma because of financial strain and neglected hip dislocations are not uncommon [7]. The current literature is limited on this subject.

Common clinical manifestations of neglected hip dislocation are shortening of the limb and limited function of the hip joint. For patients with neglected fracture, callus formation and adhesions caused by connective tissue make regular reduction difficult. The common treatments reported in previous studies for neglected hip dislocation included skeletal traction and open reduction. These treatments are associated with unsatisfactory outcomes and a high incidence of post-operative complications such as nerve injury, AVN, and osteoarthritis [8, 9]. Garrett et al. recommended total hip replacement (THR) for hip dislocations with a duration of > 3 months [10], but others have reported that limited correction of limb length discrepancy may be achieved with THR [11, 12]. Considering that the best treatment for neglected hip dislocation remains controversial and to improve clinical outcome, we propose a new surgical strategy to treat neglected hip dislocations using external fixators for preoperative traction. The purpose of this study was to assess the utility of external fixation as a means for reducing the hip joint prior to second stage open reduction and internal fixation.

## Patients and methods

We reviewed all patients with neglected hip dislocation treated in our department from January 2010 to February 2018 and identified twenty patients. Inclusion criteria were: (1) neglected hip dislocation of which duration from trauma to surgery was more than 3 weeks, (2) treated with external fixators, (3) 18 < age < 60. Seven patients were excluded because they were treated by skeletal traction or other surgery. In total, thirteen patients were enrolled in this study. Permission for this retrospective study was obtained from the Medical Ethics Committee of the authors’ institution, and written informed consent was obtained from every patient. All patients suffered posterior dislocation and the classification has been described by Thompson and Epstein [13]. The associated acetabular fracture has been classified by Judet-Letournel [14]. The function of the hip joint was evaluated using the modified Merle D’Aubigné and Postel scoring system [15], and clinical outcome was graded as follows: excellent (18), good (15–17), fair (13–14), and poor (< 14). Residual pain was assessed according to the visual analogue scale (VAS). Leg length was measured from the umbilicus to the medial malleolus by clinical examination. Anticoagulation was used from the time external fixation was performed until patient was able to begin mobilization or get out of bed. Patients began weight bearing 3 weeks after second-stage open reduction. Statistical testing was performed with the unpaired t-test, and statistical difference was defined as *P* < 0.05.

### Surgical procedures

**Fig. 1:**
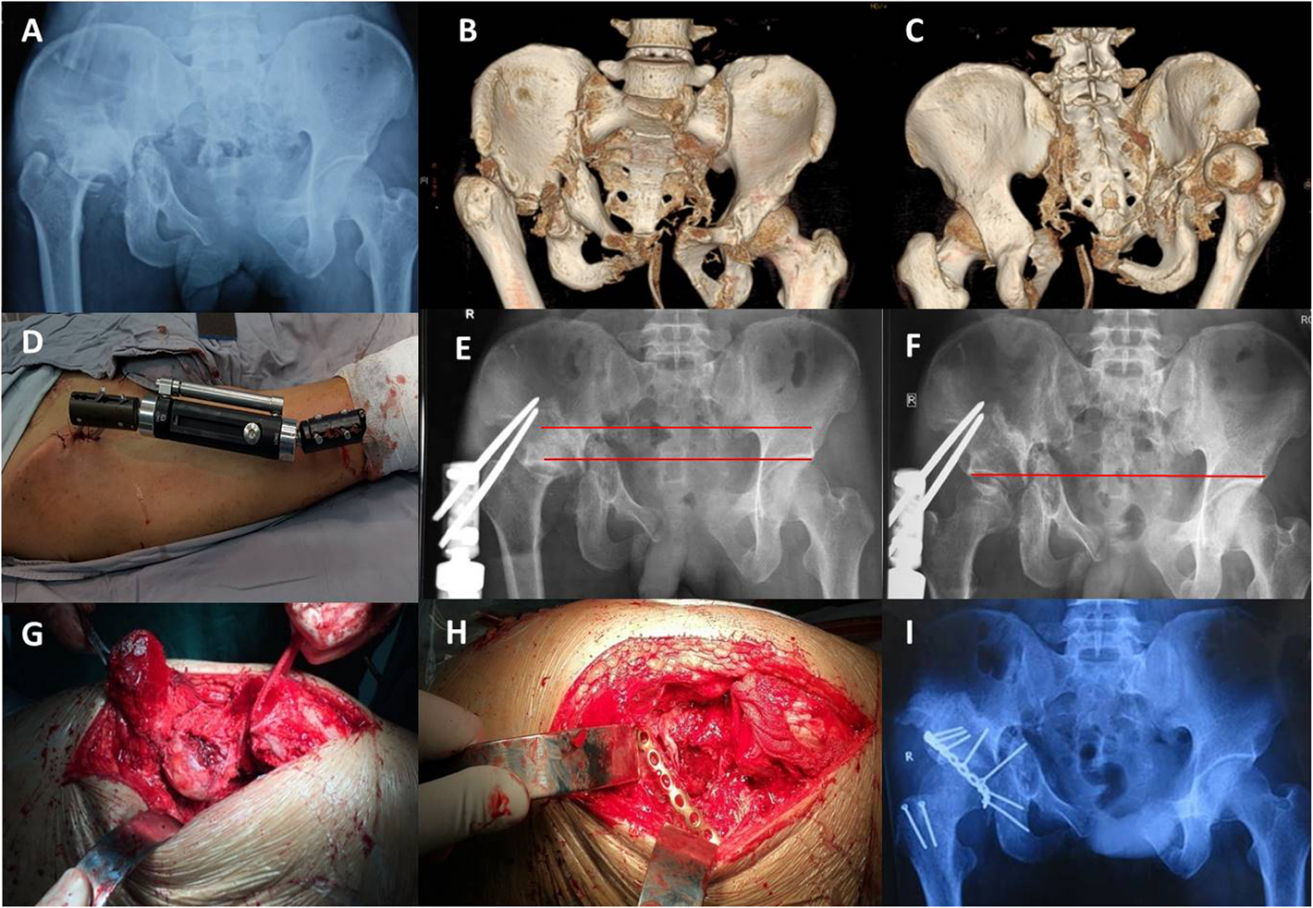
Patient 12. **a**-**c** Preoperative X-ray and three-dimensional reconstruction computed tomography images 6 months after injury. **d**-**e** External fixation placement. **f** The femoral head was drawn underneath the articular surface after 31 days of traction. **g**-**h**, **k**-**l** incision, open reduction with osteotomy of the greater trochanter, and fixation of posterior wall of acetabulum. **i** Postoperative X-ray image showed correction of limb length discrepancy and reduction of femoral head was originated from our previous study [16] and was authorized by Chinese Journal of Orthopaedic Trauma

All surgeries were performed by a single experienced orthopedic surgeon. First-stage traction by external fixation: surgeries were performed on patients under regional anesthesia. Two incisions of approximately 1 cm were made on the iliac crest and lateral thigh. Two screws were placed in the anterior inferior iliac spine, and another two screws were placed in the femur mid-diaphysis under fluoroscopic guidance. A unilateral external fixator was then connected (Fig. 1). The traction procedure began 3 days after surgery, with the external fixator stretching 1–3 mm a day. A plain radiograph was taken every 5–7 days to examine the reduction. Traction was ceased when the femoral head was drawn beneath the articular surface of the acetabulum.

Second-stage open reduction: after the first-stage traction, open reduction was performed under general anesthesia. A Kocher-Langenbeck (K-L) approach or combined ilioinguinal and K-L approaches were utilised. After superficial and deep dissection, a trochanteric flip osteotomy was performed when the acetabular roof fracture was present to allow for adequate exposure of the hip capsule. Capsulotomy was then performed, followed by clearing of fibrous tissue and intra-articular fragments, and finally a reduction of the femoral head. The malunion of posterior acetabular wall was then reconstructed and fixed with cannulated screws and a 3.5 mm reconstruction plate. An autogenous iliac bone graft was used when needed. The osteotomized trochanter was fixed with 2–3 small fragment cortex screws. Post-operative skeletal traction was used depending on the stability of fracture fixation after open reduction.

## Results

Thirteen patients (five women, eight men) with an average age of 36.7 years (range, 19–49 years) were enrolled in this study. Nine patients had associated acetabular fractures, and the pattern of fracture and posterior dislocation are shown in Table 1. The mean duration from injury to surgery was 5.0 months (range, 2.5–9 months). The mean preoperative leg-length discrepancy was 7.7 cm (range, 5–12.2 cm). Six patients presented with a postoperative leg-length discrepancy within 2 cm (Table 2). The average traction duration of external fixators was 28.8 days (range, 17–46 days). The mean operation time was 2.7 ± 0.8 h, and the average blood loss during the surgery was 1067.4 ± 374.5 mL. All patients were followed up for at least 12 months, with a mean follow-up duration of 15.4 months (range, 12–18 months). The mean Merle d’Aubigné score improved significantly from 5.1 ± 1.7 to 14.4 ± 2.0 after surgery (*p* < 0.01). The mean preoperative VAS score was 4.3 ± 1.3, and the mean postoperative score was 0.9 ± 1.0 (p < 0.01), with five patients completely free of pain. No neurovascular injuries related to limb-lengthening occurred in these patients. Complications occurred in three patients during the follow-up, two patients had AVN, and one patient had osteoarthritis of the hip joint. None of the patients received further treatment at the last follow-up.

**Table 1:** Patients’ detail

Patient	Duration from trauma to surgery(months)	Acetabular fracture type	Posterior dislocation type	Follow-up (months)	Clinical outcome	VAS	Complication
1	5	No	I	15	Good	1	
2	4.5	No	I	13	Good	0	
3	9	No	I	18	Fair	1	
4	5.5	No	I	14	Good	0	
5	3	Posterior wall	I	15	Excellent	0	
6	8	Posterior wall	II	17	Poor	2	AVN
7	4	Transverse with posterior wall	II	17	Fair	3	AVN
8	3	posterior wall	II	15	Good	0	
9	2.5	Posterior column with posterior wall	III	18	Poor	2	
10	2	Posterior wall	III	15	Good	0	
11	7	Posterior wall	III	14	Poor	1	OA
12	5	Transverse with posterior wall	IV	13	Good	1	
13	6	Posterior column with posterior wall	IV	16	Fair	1	

*AVN* Avascular femoral head necrosis

*OA* Osteoarthritis

**Table 2:** Correction of limb discrepancy

Patient	Inequality of lower limb(cm)		Traction duration(days)
	Preoperative	Postoperative	
1	5.6	0	17
2	5.4	0	21
3	8.2	1	27
4	5	0	25
5	7.5	0	25
6	8.8	1.5	34
7	12.2	2	46
8	6.0	0	27
9	9.4	2	35
10	5.3	0	20
11	11	1	38
12	7	1.5	31
13	8.2	0	28

## Discussion

Neglected hip dislocations often occur in children and are rarely reported in adults. This study showed effective correction of limb length discrepancy and significant functional improvement with external fixation-assisted reduction in thirteen adult patients. Though skeletal traction is important in joint reduction, the acetabulum becomes filled with fibrous tissue in neglected dislocations would make reduction difficult by simple traction. Skin traction usually carries a weight under 5 kg. Halo-femoral and tibial tubercle traction can carry more weight but it often ends with unsatisfactory results in patients with neglected hip dislocation [9, 17]. Prolonged dislocation may cause severe joint contracture so it is difficult to achieve satisfactory reduction by regular traction. Furthermore, heavy traction may lead to neurovascular complications.

Common complications of traumatic hip dislocation include infection, sciatic nerve palsy, AVN, heterotopic ossification, and post-traumatic arthritis [18]. Previous studies have reported that the incidence of arthritis was 16–30%, and 8.1–10% for AVN in the mid-to-long-term follow-up [1, 19]. The blood supply to femoral head is often damaged by traumatic dislocation, especially the medial femoral circumflex artery. Vascular compromise leads to intravascular coagulation and ischemic necrosis, resulting in chondral failure and accelerated joint degeneration [20]. A high incidence of osteonecrosis has been reported from 10 to 25% in hip dislocations [21]. Furthermore, the severity of the injury and time to reduction are associated with increased risks of osteonecrosis [20]. The rate of osteonecrosis is only 10% in adults after simple dislocation, but reaches 70% if the dislocation is accompanied by severe bony destruction such as fracture of the femoral head and acetabulum [22]. Hougaard and Thomsen reported that the rate of osteonecrosis of the femoral head can be reduced from 58 to 4.8% if hip reduction is performed within the first 6 h following injury [23]. Others have concluded a trend for decreased osteonecrosis of the femoral head when hip reduction was performed within 12 h [1]. In our patients, AVN occurred in 2 patients (15%), and osteoarthritis occurred in 1 patient (7.7%). All 3 patients suffered acetabular fractures with an average duration from injury to surgery of 6.3 months (range, 4 to 8 months). Patient 6 (Table 1) showed AVN 10 months after the surgery, and the Merle d’Aubigne score was improved from 4 to 12. Patient 7 (Table 1) showed AVN 12 months after surgery, and the Merle d’Aubigne score was improved from 5 to 13. Patient 11 (Table 1) showed osteoarthritis at the last follow-up (14 months), and the score was improved from 4 to 11. Though complications occurred, patients demonstrated significant functional improvement. Previous studies have also reported that complications continue to occur within 5 years [7, 24], though our study only reports a short-term follow-up result within 18 months. As the incidence of secondary complications may increase with time [25], an accurate complication rate should be recalculated for long-term follow-up.

THR has been recommended for hip dislocations with a duration of more than 3 months [10], but usually provided limited correction of limb length discrepancy within 6 cm [11, 12] and muscle release may be needed for improved correction [26]. It may provide limited efficacy for patients in this case series which all suffered limb inequality of more than 5 cm. Considering all patients were under 50 years and no signs of necrosis or arthritis occurred when on admission, we decided to perform open reduction and fixation after traction. Follow-up results showed significant pain relief and function improvement. The satisfactory rate of clinical outcome was 53.8% (excellent and good results). Limb length discrepancy was effectively corrected, and no patient was left with > 2 cm of inequality, thereby laying the foundation for possible THR in the future. AVN and arthritis occurred in three patients. Though they showed unsatisfactory outcomes (two poor and one fair), none of them received further treatment at the last follow-up. A further study evaluating the long-term clinical outcomes of external fixation with delayed open reduction against total hip replacement is required.

From our experiences, external fixation was effective for pre-reduction in patients suffering from neglected hip dislocations with limb length discrepancy, but still several contraindications are noteworthy. First, this treatment option should not be used in patients with heterotopic ossification, which most commonly occurs in the hip joint, and the incidence after traumatic dislocation was 32 to 37% [27, 28]. Traction would be resisted by ectopic bone. As the fixation technique requires stable anchors, it is unfit for patients with an unstable pelvic ring or femur. Furthermore, patients with osteoporosis should not be considered because osteoporotic bone may fail under power of traction.

There are limitations in this study including the retrospective design and a small number of patients. This study did not evaluate long-term clinical outcomes or compare the treatment strategy with other techniques. Further studies are required for this purpose.

## Conclusions

This retrospective review of 13 patients with neglected hip dislocation demonstrates the utility of a two-stage treatment regime, consisting of initial external fixation to reduce the hip joint followed by definitive open reduction and internal fixation of any associated fractures. This treatment regime resulted in improvements in leg length discrepancy, hip function and pain scores at a mean short-term follow-up of 15.4 months. Potential complications included avascular necrosis of the femoral head and post-traumatic osteoarthritis. Further studies are required to evaluate the treatment protocol against other options such as total hip replacement with long-term follow up.

## Data Availability

The data used in the current study are available from the corresponding author on reasonable request.

## Abbreviations

AVN: Avascular necrosis of the femoral head
K-L: Kocher-Langenbeck
THR: Total hip replacement
VAS: Visual analogue scale
